# Cross-Sectional Study of the Anthropometric Profile and Nutrient Status of Elite Female Ice Hockey Players: Differences by Play Position

**DOI:** 10.3390/nu16040471

**Published:** 2024-02-06

**Authors:** María José Jiménez-Casquet, Javier Conde-Pipo, Ignacio Valenzuela-Barranco, Raquel Rienda-Contreras, Fátima Olea-Serrano, Margalida Monserrat-Mesquida, Josep A. Tur, Cristina Bouzas, Miguel Mariscal-Arcas

**Affiliations:** 1 Health Science and Nutrition Research (HSNR-CTS1118), Department of Nutrition and Food Science, School of Pharmacy, University of Granada, 18071 Granada, Spainjaviercondepipo@gmail.com (J.C.-P.); folea@ugr.es (F.O.-S.); mariscal@ugr.es (M.M.-A.); 2 Cetursa Sierra Nevada, Sierra Nevada-Monachil, 18196 Granada, Spain; 3 Department of Sports and Women, Andalusian Federation of Winter Sports (FADI), 18008 Granada, Spain; raquel.rienda@gmail.com; 4 Instituto de Investigación Biosanitaria de Granada (ibs.GRANADA), 18012 Granada, Spain; 5 Research Group on Community Nutrition and Oxidative Stress, Laboratory of Physical Activity Sciences, University of Balearic Islands-IUNICS, 07122 Palma de Mallorca, Spain; margalida.monserrat@uib.es (M.M.-M.); pep.tur@uib.es (J.A.T.); 6 Centro de Investigación Biomédica en Red Fisiopatología de la Obesidad y la Nutrición (CIBEROBN), Institute of Health Carlos III, 28029 Madrid, Spain; 7 Health Research Institute of the Balearic Islands, 07120 Palma de Mallorca, Spain

**Keywords:** ice hockey, elite players, female players, nutritional status, dietary intake

## Abstract

Both the characteristics of ice hockey and the environmental conditions in which it is played affect the maintenance of the player’s nutritional status and, therefore, their state of health and performance. The primary aim of this work was to examine the anthropometric profile, estimated energy expenditure, and macronutrient and micronutrient dietary intake of elite female ice hockey players by play position. As a secondary aim, their dietary intakes were compared with the recommendations. Hypotheses suggest variations in body composition based on ice hockey players’ positions, with the expectation that these athletes may not align with energy and nutrient recommendations. Fifteen elite female ice hockey players were anthropometrically measured, basal metabolic rate and total energy expenditure were estimated, a 3-day, 24 h recall questionnaire was registered, and the results were compared with the recommended dietary intake for the Spanish population. Each player’s position on the field requires an individualized physical and nutritional approach. There are no significant imbalances (*p* > 0.05) between energy and nutrient intake in relation to the Recommended Daily Intake (RDI); however, increasing the consumption of vegetables and seafood while reducing meat and fat intake would assist these athletes in maintaining a healthier energy balance, optimizing body composition, and aligning with nutritional parameters that are better suited to enhance physical performance.

## 1. Introduction

Ice hockey is an interval team sport, with intermittent bouts of high intensity followed by longer-duration bouts of passive rest [1,2,3], requiring both aerobic and anaerobic metabolism as energy sources. To meet the energy demands and nutritional needs of an intense training regimen, the female field hockey player needs a well-chosen diet that provides the energy necessary for optimal physiological functioning and includes a wide variety of foods rich in vitamins and minerals [3,4]. Furthermore, modern ice hockey is performed at high speed and is considered one of the most contact sports, with a high risk of injury, including concussion [5]. Players are characterized by both muscle strength and endurance, muscle power, agility, and speed of movement. Adequate energy intake (EI) and carbohydrate (CHO) consumption are necessary to fuel these systems [3,6]. By play position, wingers and centers are the players who perform the greatest numbers of high-intensity movements per minute, and defenders are those who spend more accumulated time on ice and cover the most distance [7,8].

Optimization of body composition promotes optimal performance in ice hockey players [4,9,10,11]. Additional lean body mass contributes to power generation and speed, and reduced fat mass decreases frictional resistance during skating, so it is of great importance to assess body composition with accuracy and precision [12]. Indeed, talent identification processes have been developed based on the assessment of anthropometric profiles, which vary according to play position [2,10,11]. It seems logical to think that the distribution of roles in the field is based on skills and physical conditions, which in turn are conditioned by anthropometric characteristics [10,13,14,15].

However, unlike other team sports, research on this topic remains scarce among female athletes, focused mainly on males from other sports leagues in other countries, and the results are controversial [11,15]. One of the obstacles facing hockey researchers cross-culturally lies in the fact that Europeans and North Americans play ice hockey in considerably different conditions. More specifically, European ice hockey is played on international-sized ice surfaces, while North American ice hockey is played on NHL-sized ice surfaces. At first glance, this may not seem like a substantial difference, but in a game where physical contact and a confined playing surface are believed to be at least partly responsible for aggressive behavior. In addition, the Europeans are governed by international ice hockey rules, which include differences in the use of the red line and also in the degree to which certain infractions are penalized. For example, fighting is almost totally absent from European hockey, while it is relatively frequent in North American hockey. According to the European rules, athletes who fight will receive a 10 min penalty for unsportsmanlike conduct, whereas the North American NHL rules only stipulate a 5 min penalty. However, these external and confounding factors have made direct comparisons between NHL and US rules inconclusive [16].

Thus, whereas some authors such as Schulze et al. [13] or Sigmund et al. [17] found greater values in height, body mass, or body fat in male defenders, Wörner et al. [18] observed greater height and lower weight in goalkeepers, and Triplett et al. [19] found higher body fat in goalkeepers. Vigh-Larsen et al. [2] found statistically significant differences in body mass and muscle but not in body fat between elite and sub-elite players and no differences between play positions. Lemoyne et al. [14] observed differences related to play position in the anthropometric profile of males but not in females, exactly the opposite of Geithner et al. [20].

The hydration status of female hockey players must be considered for performance optimization. Although indoor ice hockey is performed in cold and humid environments (~5–10 °C), which may decrease the importance of adequate hydration, it is known that these athletes produce high sweat rates, with the consequent loss of water, sodium, and other electrolytes, particularly during match sessions [21,22,23], that can produce dehydration in the athlete. Despite the numerous opportunities to drink while resting during game time [24], players lose a high percentage of their body mass (~2–8%) as a result of mild dehydration [7,21,25]. This can be attributed to the rigorous physical demands inherent to this sport and to wearing heavy protective equipment that also hinders transpiration and heat dissipation [23,26]. Mild dehydration in ice hockey players is associated with dangerous increases in core temperature, which induce central fatigue, negative athletic performance, and increased muscle glycogen utilization [7,22,23,27,28]. This muscle glycogen should be readily disposable for its primary sports need, which is to maintain performance and offset fatigue. Carbohydrate is the most important fuel for ice hockey players, with daily intakes observed in male adolescents of ~9–11 g/kg [29] and ~2.5–7.6 g/kg in varsity females [3]. Women have lower whole-body sweat rates and sweat sodium concentrations than men, but in most cases, these differences are attributed to lower absolute workloads. During the luteal phase, there is an increase in the core body temperature threshold for the onset of sweating, but there are no differences in whole-body sweat rate across the phases of the menstrual cycle. Female hockey players should drink enough fluid during exercise to avoid significant hypohydration, as 2% body mass loss is associated with performance decrements in women, and should achieve an adequate intake of 2 L per day or 20.9 mL every 15 min [3,7,21,22,23,24,25,26,27,28,29].

Hence, the inherent characteristics of ice hockey and its environmental conditions have nutritional implications for maintaining energy, carbohydrate availability, and fluid balance, which could affect health status and performance [12,26]. Nonetheless, team sport athletes, including ice hockey players, do not usually meet their dietary intake recommendations and report eating disorders such as restrictive eating, low energy intake, or nutrient deficiencies. This problem was exacerbated in female athletes due to a sports culture that was very focused on aesthetics [3,30].

On the other hand, it cannot be ignored that unhealthy dietary behaviors, such as high intakes of fast food and sugar-sweetened beverages, are prevalent among female adolescents, even in those who participate in organized sports, including ice hockey, and neither can maturation be ignored as a condition that could probably affect the body composition of female athletes [23,31,32]. Furthermore, the sports facilities in which ice hockey is practiced are frequently oriented toward recreational use, and for this reason, they are located in large commercial areas where fast-food restaurants abound. Thus, an environment of high exposure to unhealthy food is created, which has a great influence on the eating behavior of these female adolescent ice hockey practitioners [23].

Several studies have shown that female athletes are at increased risk for macronutrient and micronutrient deficiencies [3,9,10,11,12,13,14,15,16,17,18,19,20,21,22,23]. While proper nutrition is a key component of health and performance, female athletes are at an increased risk of nutrient deficiencies, eating disorders, and dietary-related health issues compared to their male counterparts. We hypothesized that there are differences in body composition by play positions and that these athletes would not meet the energy and nutrient recommendations. Therefore, the primary aim of this work was to examine the anthropometric profile, estimated energy expenditure, and macronutrient and micronutrient dietary intake of elite female ice hockey players by play position. As a secondary aim, we also compared their dietary intake with the recommendations.

## 2. Materials and Methods

The study design was cross-sectional, descriptive, and comparative.

### 2.1. Ethics

The study protocols and procedures were developed in accordance with the standards of the Declaration of Helsinki and approved by the Research Ethics Committee of the University of Granada, Spain (ref. 1162/CEIH/2020). Prior to participating in the study, all participants were informed of the objectives of the research and provided their written informed consent.

### 2.2. Participants

The sample comprised 15 ice hockey players (21.40 years (SD: 6.50)) from an elite Spanish female team. The inclusion criterion was being an active team member. Players who were injured or ill during the study were excluded. Data were collected during the competition season. The players represented all play positions: goalkeeper, defender, center, and winger [16]. In some studies, the last two are grouped as forwards.

### 2.3. Procedures

#### 2.3.1. Anthropometric Variables

Anthropometric variables were taken by trained regular personnel of the European Leagues, certified by ISAK, the International Society for the Advancement of Kinanthropometry, with a technical measurement error of 0.04% for basic measurements and of 2.12% for skinfolds, following the international standards recommended by the ISAK and by Papadimitriou et al. [33].

All measures were taken in the morning. In addition, shoes, socks, stockings, or anything else that could affect the measurement were removed. The participants, during the measurement, remained relaxed in the supine position and with an empty stomach. The female athletes were in the mid-period of menstruation without any problems with menstruation [33]. Height was measured in centimeters using a wall-mounted stadiometer (Seca 214, SECA Deutschland, Hamburg, Germany), and weight was measured in kilograms with a high-precision scale (Tanita BC-418, Tanita, Tokyo, Japan). On the measurement days, the hockey players should have neither performed high-intensity exercise the previous day nor performed training or stretching sessions on the same day. All participants were weighed while wearing light clothing and barefoot (0.6 kg was subtracted from the total for clothing) [33,34].

Body mass index (BMI) was calculated by dividing the weight in kilograms by the square of the height in meters (kg/m^2^). The skinfolds (bicipital, tricipital, subscapular, suprailiac, abdominal, mid-leg, and thigh) were measured with a Holtain plicometer (Holtain Ltd., Crymych, UK). Faulkner’s fat estimation formula was used [35]. All anthropometric measurements were taken in triplicate, using the mean of both for subsequent analysis.

#### 2.3.2. Estimated Energy Expenditure

Basal metabolic rate (BMR) was estimated through the Harris–Benedict formula [36]. Total energy expenditure (TEE) was estimated following the formula proposed by FAO, WHO, and United Nations University [37] based on BMR. A physical activity level (PAL) factor of 1.82, specific for women, was applied for the final estimate.

#### 2.3.3. Dietary Assessment

The dietary assessment method utilized was a 3-day, 24 h recall questionnaire (R24h), previously validated by the research group [38]. This questionnaire captures the dietary habits of the participants over the past few days and allows for the estimation of their food intake, energy consumption, and essential nutrient intake. Trained interviewers conducted face-to-face individual interviews to gather this information.

The third questionnaire spanned three non-consecutive days, including one non-working day, covering a full 24 h each day [39,40]. Subsequently, the intake of essential micronutrients was compared to the Recommended Dietary Intake (RDI) for the general female population in Spain [41,42], as specific recommendations for individuals involved in ice hockey or athletes in general were not available. To calculate the average RDI, which varies according to age, these micronutrient recommendations were considered. Questionnaires were assessed for reliability based on predefined cutoff points, following the criteria established by Goldberg et al. [39,40,43]. These criteria considered the results of energy intake, daily energy expenditure (as determined by the three-day recall), and basal metabolic rate (BMR) [37,44].

All the female athletes under study were classified and analyzed by positions (defender *n* = 3, center *n* = 5, winger *n* = 5, goalkeeper *n* = 2) as the objective of the study and due to their specificity of match performance [1,2,3,4,6,7,8,9,10,11,12,13,14,15,16,17,18,19,20].

### 2.4. Statistical Analysis

Nutritional intakes were estimated using the Dial program (2015 Alce Ingeniería) in combination with the AUSNUT 2011-13 food nutrient database. Statistical analysis was performed with the statistical computing software R (v 4.1.2., R Core Team, Vienna, Austria). The normality of the variables was analyzed using the Kolmogorov–Smirnov test with the Lillieforts correction, and homoscedasticity was analyzed with the Levene test. Means and standard deviations were used for the basic descriptions. For the comparisons between groups of continuous variables, the nonparametric tests for independent variables, Welch’s ANOVA, were used to calculate the effect size and the eta-square index. For multiple comparisons, the Games–Howell post hoc test was used. In the case of bivariate correlations, Spearman’s rho correlation coefficient was used. All reported p values are based on the two-tailed test, and the level of statistical significance for all tests was set at 95%.

## 3. Results

**Figure 1:**
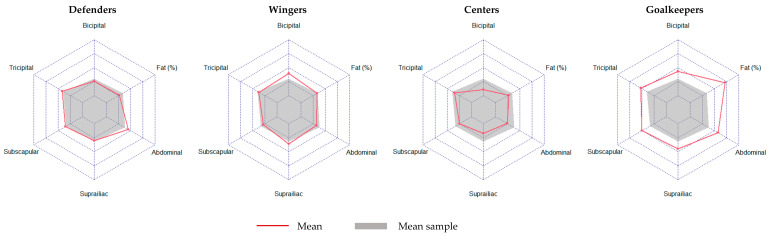
Radar chart of main anthropometric measures by play positions.

**Figure 2:**
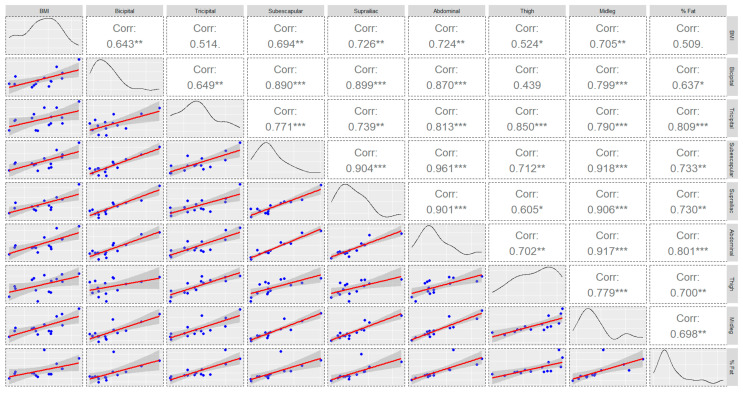
Chart of correlations, density curve, and regression models of anthropometric measures of the sample (* *p* ≤ 0.050; ** *p* ≤ 0.010; *** *p* ≤ 0.001).

Table 1 and Figure 1 show a comparison of the anthropometric characteristics by play positions. Statistically significant differences were found between positions in the suprailiac fold (*p* = 0.002; *η*^2^ = 0.82; CI = 0.33–1.00), abdominal fold (*p* = 0.002; *η*^2^ = 0.91; CI = 0.52–1.00), and leg (*p* = 0.045; *η*^2^ = 0.58; CI = 0.00–1.00), but not in the percentage of fat (*p* = 0.504; *η*^2^ = 0.34; CI = 0.00–1.00). The highest means corresponded to goalkeepers (suprailiac and abdominal fold) and to wingers (midleg). Bivariate correlations between these characteristics are shown in Figure 2. For the percentage of fat, the highest correlation values were found in the tricipital (r = 0.809) and abdominal folds (r = 0.801).

As shown in Table 2, only goalkeepers and wingers had an energy intake (EI) that matched their total energy expenditure (TEE). There were only statistically significant differences between groups in intake expressed in grams for carbohydrates and soluble fiber. The highest mean intakes of carbohydrates corresponded to goalkeepers (*p* = 0.003; *η*^2^ = 0.86; CI = 0.37–1.00) and of soluble fiber to wingers (*p* = 0.043; *η*^2^ = 0.10; CI = 0.00–1.00). For the whole sample, the caloric profile was 39.88% from lipids, 37.33% from carbohydrates, and 20.20% from protein, without statistically significant differences by play positions. Water and beverage fluid intake averaged 3229.73 mL/day. These data could probably explain the level of the sample not following a structured dietary plan.

**Figure 3:**
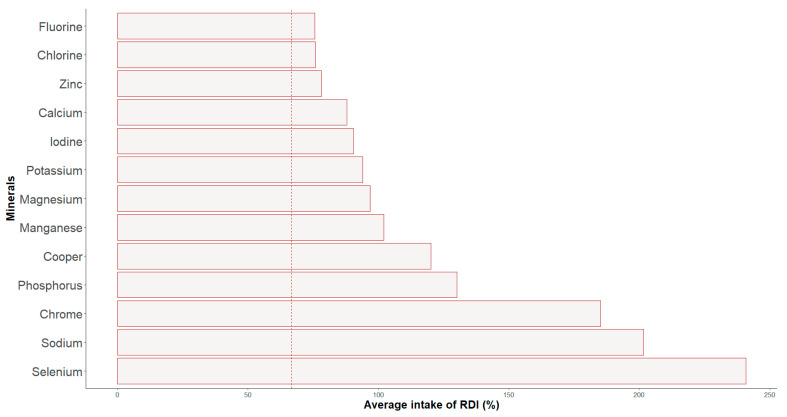
Percentage of adjustment to the RDIs of minerals’ intakes.

The daily mineral intake and percentage of RDI by play position are shown in Table 3 and Figure 3. The results revealed the highest intakes of iron (*p* = 0.013; *η*^2^ = 0.73; IC = 0.00–1.00) in wingers and of iodine (*p* = 0.001; *η*^2^ = 0.95; IC = 0.79–1.00), magnesium (*p* = 0.023; *η*^2^ = 0.67; IC = 0.00–1.00), and phosphorous (*p* = 0.007; *η*^2^ = 0.65; IC = 0.00–1.00) in goalkeepers. For the whole group, the mean intake of all the minerals was more than 2/3 of the IDR.

**Figure 4:**
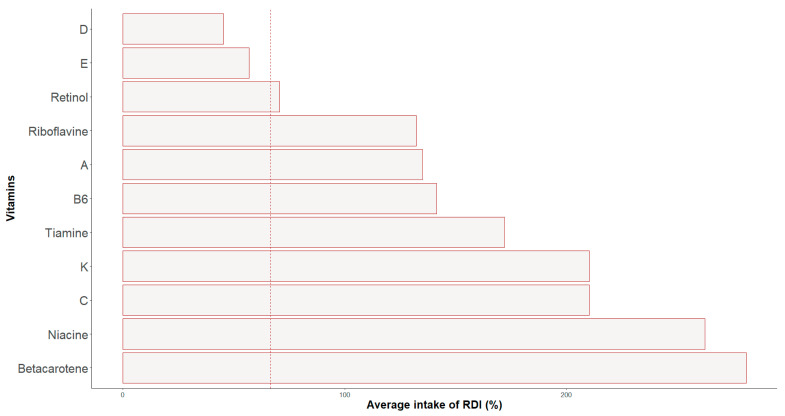
Percentage of adjustment to the RDIs of vitamins’ intakes.

Regarding the vitamin daily intakes (Table 4, Figure 4), only vitamin A (*p* = 0.028; *η*^2^ = 0.62; IC = 0.00–1.00) and retinol (*p* = 0.028; *η*^2^ = 0.67; IC = 0.00–1.00) showed statistically significant differences between positions, with the highest values in wingers. For the whole group, the mean intakes of vitamins D and E were less than 2/3 of the RDI.

## 4. Discussion

This research work presents data that could expand knowledge about anthropometric characteristics, estimated energy expenditure, and dietary intake of macronutrients and micronutrients in Spanish elite female ice hockey players by play position, comparing their nutrient intakes with the recommendations. We hypothesized that there must be differences in body composition by play positions, finding that not all of them are statistically significant and that these female athletes do not follow all the recommendations for energy and nutrient intake.

The goalkeepers were the group with the highest height, weight, and percentage of body fat, followed by the defenders, wingers, and centers, results that are consistent with other studies that also focused on elite female ice hockey teams [19,44]. These findings are also similar to those of earlier studies conducted on male ice hockey teams, although these did not include goalkeepers [2,13,17,18,19].

Considering the whole sample, the fat percentage values were much higher than those reported by Gilenstam and Geithner [12], probably because X-ray absorptiometry (DXA) is a precise tool while skinfold measurements are an operator-dependent method. Regarding fat distribution, goalkeepers were the players who accumulated a higher percentage of fat in the abdominal area, which may be due to the secondary role of the upper body for goalkeepers, which involves a less muscular implication (15).

The caloric expenditure of each position can influence body composition due to the specific performance of each position [45]. This makes it important to assess nutritional needs and training per subject and per position [46]. Our results provide useful female reference values for coaches and physical trainers that could add interest and improve their testing protocol for the talent identification process, as detailed by Lemoyne et al. [14].

Using female hormones and biomarkers can provide information to coaches, athletes, and sports scientists on the physiological response to training and provide additional insight into a female athlete’s readiness to maximize player health and performance [47,48,49].

To optimize adaptations to training, enhance sports performance, and promote rapid recovery, athletes must match energy intake (EI) with total energy expenditure (TEE) [50]. Nevertheless, in our study, the EI’s of both the defenders and the centers were not sufficient to cover the estimated TEE. The greater need for agility and speed, as well as aerobic and anaerobic power, of field players compared to goalkeepers may be behind their lower EI [20]. However, this situation could also affect their health status, increasing the risk of low energy availability [50,51,52], whose threshold has recently been placed for female athletes at an intake less than or equal to 30 kcal/kg FFM/day [53]. It also increases the risk of the female athlete triad, which presents clinical manifestations that include eating disorders, hypothalamic functional amenorrhea, and osteoporosis [54,55].

The proportion of energy obtained from lipids and proteins notably exceeds the proportions recommended for the Spanish adult population [42] and those reported in other studies with female ice hockey players [29] or female athletes in general [56], which could probably explain the level of the sample not following a structured dietary plan. Because during an ice hockey match muscle glycogen declines between 38 and 88%, the low proportion of carbohydrates could affect physical performance [1,57].

The high proportion of lipids and proteins consumed may be due to the abandonment of the Mediterranean diet that is currently occurring among young Spaniards, with serious consequences for health [34]. Indeed, the elevated levels of cholesterol and saturated fatty acids (SFA) can be attributed to a dietary pattern that emphasizes meat consumption at the expense of vegetables. The inconsistency between energy intake and the high percentage of body fat in all of the groups, with the exception of goalkeepers, could be attributed to issues such as irregular meal schedules, energy imbalances, or improper distribution of calorie intake and denotes a lack of education in nutrition [58].

The typical daily replenishment of water through the consumption of food and beverages is approximately 4% of body weight in adults as a general guideline [41]. While this figure appears to be achieved by all groups except for goalkeepers and the water intakes reported are in line with those reported by Vermeulen et al. [29], it is important to consider that water losses during ice hockey training range from 1.3 to 4.3% of body weight [24,27,59]; therefore, daily water intake is likely insufficient and could be concealing a state of mid-dehydration. These players should heed the recommendation to maintain proper hydration by using a carbohydrate–electrolyte solution [21,27,59].

In the current study, most vitamins and minerals were adequately consumed or were within 2/3 of the RDI.

Regarding mineral intake, in the case of the central group, there seem to be deficiencies in calcium, iron, iodine, and zinc. In winter sports, low levels of iron [26] and iodine [48] are frequently reported, possibly due to reduced consumption of dairy products and seafood such as oily fish or mollusks. Other authors have also reported deficiencies in calcium and zinc among child and young adolescent ice hockey players, with consequent effects on performance [29]. Sodium intake exceeded recommended levels in all groups, once again pointing to excessive consumption of animal and processed products. Sodium is a crucial electrolyte in the body, and while substantial amounts can be lost through sweating during an ice hockey match [22], its excess is associated with adverse health effects, including elevated blood pressure and hypertension [29].

For the whole sample, only vitamin D and E intakes were lower than 2/3 of RDI. Although vitamin D is essential for bone mineralization along with calcium [50] and also plays a role in autoimmunity and metabolic function, there is a high prevalence of vitamin D globally [60], especially in athletes who practice indoor sports such as ice hockey players, due to the fact that the majority of vitamin D is synthesized from the sunlight [29,60,61]. A deficit of vitamin D is also correlated with an excess of body fat [60]. This could also help to clarify the high levels of body fat found in this study. In the case of goalkeepers with notably low vitamin D levels, values representing only 20% of the RDI correspond with significantly elevated body fat percentages, approximately 27%. Therefore, it seems important for ice hockey players to increase their intake of products rich in vitamin D, such as oily fish, liver, and eggs [50].

Vitamin E is another antioxidant that plays a significant role in athletes because of the production of free radicals during exercise [62]. In order to maintain adequate levels of vitamin E, it is necessary to consume nuts, seeds, and green leafy vegetables (such as spinach and broccoli), all of which are components of the Mediterranean diet and, therefore, easy to consume by the population studied [32,63].

The high consumption of vitamin B_12_ through dietary food intake, exceeding double the RDI, reflects an excess of animal-derived fatty foods in the diet of these athletes, along with the high levels of cholesterol, protein, and saturated fat intake [64].

Finally, considering the fluctuating hormone levels during the menstrual cycle, it is crucial to address the nutritional requirements of female athletes, who may have elevated needs for zinc during the mid-luteal phase and for folate, riboflavin, and B_12_ in the follicular phase [65].

### Strength and Limitations

The primary strengths of this study lie in its pioneering analysis of a Spanish professional women’s hockey team, combining anthropometric data with nutritional insights to provide a comprehensive perspective. The current study also has limitations. Notably, it features a descriptive cross-sectional design and employs a sample size that precludes the establishment of causal inferences.

Another obstacle is the cross-cultural difference in European and North American ice hockey playing conditions faced by ice hockey researchers.

Therefore, it is essential to interpret these findings with caution. First, dietary habits relied on self-reporting by the participants, and prior studies involving female athletes have highlighted the issue of underreporting of dietary intake in self-reported data [3]. Second, the current study makes certain assumptions when predicting average daily energy expenditure. To enhance the accuracy of these estimates, the use of more precise measures, such as doubly labeled water, heart rate monitoring, or accelerometers, would be advisable. The fat percentage values were much higher than those of other authors, probably because they used dual-energy X-ray absorptiometry (DXA) instead of skinfold measurements. Additionally, the RDI for micronutrients utilized in this study is based on estimates for the general Spanish female population, with adjustments made based on the average RDI across the age range of these athletes. This approach may potentially lead to an underestimation of specific RDIs, highlighting the need for tailored RDIs specifically designed for ice hockey athletes. It should be noted that menstrual cycle issues such as amenorrhea or oligomenorrhea have not been considered.

## 5. Conclusions

These data present an anthropometric and nutritional profile for a group of female athletes who have not been previously studied: elite female ice hockey players from the Spanish First Division of Ice Hockey (Iberdrola League). These results provide high-performance female sports data that are useful for coaches and physical trainers and may help in the talent identification process, as well as assist in the nutritional health care of female hockey players. Results seem to indicate that each position in the field requires an individualized physical and nutritional approach, although further research is needed. While there appear to be no significant imbalances between energy and nutrient intake in relation to the nutritional recommendations, there is a decrease in adherence to the Mediterranean Diet. The Mediterranean diet is a model for a balanced, equilibrated, and healthy diet deeply rooted in Spanish culture. Increasing dietary intake of vegetables and seafood while reducing dietary intake of meat and fat would help these female athletes maintain a healthier energy balance, optimize body composition, and align with nutritional parameters that are better suited to enhance physical performance, promoting nutritional health that could prevent sports injuries.

## Figures and Tables

**Table 1:** Anthropometric characteristics of the study sample by play position.

Variable (Mean, SD)		Play Position				Sig.	Effect Size	
	Sample (*n* = 15)	Defender (*n* = 3)	Center (*n* = 5)	Winger (*n* = 5)	Goalkeeper (*n* = 2)	*p*	*η*²	IC
Height (cm)	163.47 (8.07)	167.80 (5.89)	155.33 (8.33)	161.60 (7.92)	169.50 (0.71)	0.098	0.42	(0.00, 1.00)
Weight (kg)	67.41 (13.67)	73.24 (10.08)	56.57 (11.30)	64.10 (16.82)	77.35 (7.00)	0.249	0.10	(0.00, 1.00)
BMI (kg/m^2^)	25.02 (3.37)	25.91 (2.13)	23.33 (3.13)	24.39 (4.88)	26.91 (2.21)	0.511	0.14	(0.00, 1.00)
Tricipital skinfold (mm)	20.88 (5.02)	21.02 (5.14)	20.00 (1.60)	20.45 (6.89)	22.90 (6.36)	0.410	0.11	(0.00, 1.00)
Bicipital skinfold (mm)	12.42 (5.50)	11.68 (5.77)	8.70 (2.30)	14.35 (7.01)	15.00 (2.55)	0.350	0.17	(0.00, 1.00)
Subscapular skinfold (mm)	17.20 (7.36)	17.84 (6.78)	15.00 (0.28)	16.00 (11.17)	21.90 (2.69)	0.513	0.08	(0.00, 1.00)
Suprailiac skinfold (mm)	18.66 (7.25)	18.22 (5.97)	14.50 (1.50)	20.02 (11.13)	22.60 (0.85)	0.002	0.82	(0.33, 1.00)
Abdominal skinfold (mm)	21.73 (8.05)	23.76 (8.11)	17.90 (0.50)	19.86 (11.22)	27.10 (0.99)	0.002	0.91	(0.52, 1.00)
Thigh skinfold (mm)	30.88 (6.34)	32.36 (5.71)	29.80 (4.80)	28.25 (8.37)	35.50 (3.54)	0.489	0.15	(0.00, 1.00)
Midleg skinfold (mm)	23.57 (8.07)	26.32 (8.33)	19.20 (2.40)	22.64 (11.33)	25.60 (0.57)	0.045	0.58	(0.00, 1.00)
Faulkner Fat (%)	18.69 (5.83)	18.15 (3.70)	16.10 (0.43)	17.46 (6.09)	27.02 (10.18)	0.504	0.34	(0.00, 1.00)

**Table 2:** Macronutrients daily intake by play position.

		Play Position				Sig.	Effect Size	
Variable	Sample	Defender	Center	Winger	Goalkeeper	*p*	*η*²	IC
BMR (kcal) *	1431.82 (184.08)	1503.15 (175.15)	1270.50 (123.98)	1390.00 (194.99)	1600.03 (0.74)	0.230	0.36	(0.00, 1.00)
TEE (kcal) *	2147.73 (276.11)	2254.72 (262.72)	1905.75 (185.98)	2085.00 (292.48)	2400.04 (1.11)	0.230	0.36	(0.00, 1.00)
Energy intake (kcal) *	2020.67 (514.86)	1966.80 (393.76)	1559.00 (313.93)	2085.60 (482.39)	2685.50 (635.69)	0.290	0.24	(0.00, 1.00)
Water (g) *	3229.73 (894.59)	2830.00 (741.64)	3264.33 (883.20)	3734.20 (1137.05)	2916.00 (22.63)	0.503	0.20	(0.00, 1.00)
Protein (g) *	102.13 (32.63)	94.52 (32.83)	75.43 (9.52)	113.98 (26.20)	131.55 (52.96)	0.127	0.33	(0.00, 1.00)
Carbohydrate (g) *	185.40 (47.85)	179.80 (30.28)	157.00 (42.14)	176.40 (48.32)	264.50 (2.12)	0.003	0.86	(0.37, 1.00)
Lipid (g) *	91.42 (35.95)	92.16 (37.19)	65.27(12.63)	95.96 (39.41)	117.70 (49.92)	0.374	0.13	(0.00, 1.00)
Simple carbohydrate								
Glucose (g) *	11.43 (4.92)	11.14 (5.059	14.33 (4.92)	10.50 (6.14)	10.11 (1.85)	0.700	0.10	(0.00, 1.00)
Fructose (g) *	13.90 (5.52)	12.14 (4.94)	18.70 (6.66)	13.72 (5.76)	11.55 (3.32)	0.587	0.22	(0.00, 1.00)
Lactose (g) *	14.68 (6.60)	14.04 (3.86)	10.81 (1.33)	16.06 (8.57)	18.60 (12.7)	0.425	0.11	(0.00, 1.00)
Soluble fibre (g) *	5.31 (4.90)	3.68 (1.00)	6.23 (0.55)	6.94 (8.63)	3.90 (0.85)	0.043	0.10	(0.00, 1.00)
Indissoluble fibre (g) *	9.41 (6.96)	7.04 (3.06)	12.13 (2.39)	11.64 (11.38)	5.70 (2.97)	0.191	0.15	(0.00, 1.00)
Cholesterol (mg) *	444.00 (299.56)	221.00 (87.46)	458.33 (261.40)	737.00 (375.47)	394.00 (261.63)	0.264	0.48	(0.00, 1.00)
Caloric profile								
Proteins (%)	20.20 (3.60)	18.84 (2.449	19.56 (1.62)	19.20 (3.34)	19.56 (1.62)	0.715	0.00	(0.00, 1.00)
Carbohydrates (%)	37.33 (7.16)	37.91 (9.88)	39.92 (3.78)	33.94 (5.32)	40.49 (9.27)	0.517	0.14	(0.00, 1.00)
Lipids (%)	39.88 (7.11)	41.08 (8.02)	37.80 (2.60)	40.46 (9.41)	38.54 (7.61)	0.867	0.04	(0.00, 1.00)
Lipids profile								
SFA (%)	13.63 (4.90)	16.23 (5.27)	9.13 (2.39)	13.53 (5.39)	14.15 (1.63)	0.136	0.37	(0.00, 1.00)
MUFA (%)	16.63 (3.98)	18.84 (2.44)	19.56 (1.62)	22.36 (5.14)	19.20(3.34)	0.715	0.00	(0.00, 1.00)
PUFA (%)	5.42 (2.18)	4.44 (2.32)	4.80 (1.339	7.03 (2.24)	4.81(1.45)	0.425	0.43	(0.00, 1.00)

Note: * means (SD). BMR: basal metabolic rate; TEE: total energy expenditure; SFA: saturated fatty acids; MUFA: monounsaturated fatty acids; PUFA: polyunsaturated fatty acids.

**Table 3:** Mineral daily intake and adjustment percentage to RDI by play position group.

			Play Position				Sig.	Effect Size	
Variable		Sample	Defender	Center	Winger	Goalkeeper	*p*	*η*²	IC
Calcium	Dietary intake (mg)	1123.73 (768.60)	1341.20 (1017.79)	418.00 (340.07)	1184.00 (677.36)	1488.00 (214.96)	0.047	0.56	(0.00, 1.00)
	% RDI	87.97 (60.50)	103.17 (78.29)	32.15 (26.16)	91.08 (52.10)	125.93 (32.75)	0.114	0.47	(0.00, 1.00)
Iron	Dietary intake (mg)	13.94 (7.23)	11.68 (2.05)	10.07 (0.93)	18.80 (11.43)	13.25 (0.21)	0.013	0.73	(0.00, 1.00)
	% RDI	78.44 (40.24)	64.89 (11.40)	55.93 (5.16)	104.44 (63.50)	81.06 (11.71)	0.168	0.42	(0.00, 1.00)
Iodine	Dietary Intake (µg)	105.12 (60.69)	89.78 (44.42)	53.43 (10.49)	135.32 (81.30)	145.50 (0.71)	0.001	0.95	(0.79, 1.00)
	% RDI	90.44 (52.22)	78.07 (39.63)	46.46 (9.12)	117.67 (70.69)	119.25 (10.90)	0.005	0.84	(0.26, 1.00)
Zinc	Dietary intake (mg)	10.86 (2.95)	9.60 (1.80)	8.17 (2.12)	12.74 (2.96)	13.35 (2.19)	0.183	0.40	(0.00, 1.00)
	% RDI	78.19 (34.99)	64.00 (12.01)	54.44 (14.15)	84.93 (19.75)	132.46 (76.07)	0.281	0.28	(0.00, 1.00)
Magnesium	Dietary intake (mg)	311.80 (104.87)	279.80 (51.82)	241.67 (41.65)	369.00 (158.02)	354.00 (9.90)	0.023	0.67	(0.00, 1.00)
	% RDI	96.73 (33.72)	84.79 (15.70)	73.23 (12.62)	111.82 (47.88)	124.10 (20.79)	0.176	0.41	(0.00, 1.00)
Potassium	Dietary intake (mg)	3236.40 (1012.65)	2774.80 (619.24)	2741.33 (260.23)	3940.00 (1415.58)	3374.00 (598.21)	0.400	0.09	(0.00, 1.00)
	% RDI	93.96 (30.37)	79.28 (17.69)	78.32 (7.44)	112.57 (40.45)	107.62 (32.96)	0.434	0.06	(0.00, 1.00)
Sodium	Dietary intake (mg)	3024.80 (2283.57)	3973.40, (2785	2107.33 (1349.19)	1960.80 (1288.08)	4689.50 (3669.18)	0.588	0.00	(0.00, 1.00)
	% RDI	201.65 (152.24)	264.89 (185.71)	140.49 (89.95)	130.72 (85.87)	312.63 (244.61)	0.588	0.00	(0.00, 1.00)
Selenium	Dietary Intake (µg)	116.33 (50.60)	11.16 (37.50)	73.40 (26.64)	141.40 (69.41)	131.00 (16.97)	0.161	0.34	(0.00, 1.00)
	% RDI	240.84 (110.34)	222.32 (75.00)	146.80 (53.28)	282.80 (138.82)	323.29 (120.61)	0.294	0.24	(0.00, 1.00)
Manganese	Dietary intake (mg)	2.97 (1.66)	2.82 (1.16)	2.37 (0.87)	3.34 (2.38)	3.35 (2.62)	0.886	0.00	(0.00, 1.00)
	% RDI	102.03 (53.74)	94.00 (38.83)	78.89 (29.12)	111.33 (79.36)	133.54 (56.27)	0.705	0.00	(0.00, 1.00)
Cooper	Dietary intake (mg)	1.53 (0.59)	1.32 (0.33)	1.31 (0.39)	1.80 (0.87)	1.75 (0.49)	0.640	0.00	(0.00, 1.00)
	% RDI	120.15 (45.65)	101.38 (25.35)	100.51 (30.00)	138.46 (67.28)	150.77 (15.23)	0.122	0.43	(0.00, 1.00)
Chrome	Dietary Intake (µg)	43.93 (23.68)	9.66 (5.02)	4.47 (1.61)	11.00 (11.21)	7.65 (3.04)	0.298	0.21	(0.00, 1.00)
	% RDI	185.15 (99.82)	153.92 (104.07)	149.72 (54.77)	243.25 (113.32)	171.13 (113.56)	0.624	0.33	(0.00, 1.00)
Chlorine	Dietary intake (mg)	1744.13 (779.30)	1644.20 (355.07)	1141.67 (524.03)	2050.80 (1114.44)	2131.00 (756.60)	0.488	0.47	(0.00, 1.00)
	% RDI	75.83 (33.88)	71.49 (15.44)	49.64 (22.78)	89.17 (48.45)	92.65 (32.90)	0.488	0.47	(0.00, 1.00)
Fluorine	Dietary intake (mg)	2.42 (1.04)	2.61 (1.09)	2.22 (1.03)	2.49 (1.32)	2.09 (0.86)	0.929	0.09	(0.00, 1.00)
	% RDI	75.70 (38.65)	87.07 (36.48)	73.89 (34.30)	83.13 (43.99)	31.42 (25.34)	0.314	0.52	(0.00, 1.00)
Phosphorus	Dietary intake (mg)	1510.07 (440.68)	1319.40 (140.88)	1027.00 (136.17)	1811.40 (383.55)	1958.00 (526.09)	0.007	0.65	(0.00, 1.00)
	% RDI	130.15 (47.54)	109.95 (11.74)	85.58 (11.35)	195.53 (89.61)	150.95 (31.96)	0.086	0.63	(0.00, 1.00)

Note: Data are presented as means (SD). % RDI: adjustment percentage of RDI for the general Spanish female population.

**Table 4:** Vitamins daily intake and adjustment percentage to RDI by play position.

			Play Position				Sig.	Effect Size	
Variable		Sample	Defender	Center	Winger	Goalkeeper	*p*	*η*²	IC
Thiamine	Dietary intake (mg)	1.56 (0.69)	1.72 (0.96)	1.03 (0.29)	1.59 (0.61)	1.9 (0.42)	0.228	0.29	(0.00, 1.00)
	% RDI	172 (75.69)	190.67 (106.59)	114.44 (32.72)	177.11 (67.97)	198.89 (29.86)	0.162	0.35	(0.00, 1.00)
Riboflavin	Dietary intake (mg)	1.82 (0.59)	1.51 (0.27)	1.25 (0.26)	2.24 (0.49)	2.40 (0.71)	0.116	0.55	(0.00, 1.00)
	% RDI	132.35 (47.35)	108.00 (19.52)	89.29 (18.56)	160.00 (34.85)	188.69 (74.92)	0.126	0.54	(0.00, 1.00)
Niacine	Dietary intake (mg)	38.69 (13.779	29.88 (7.13)	33.87 (7.67)	46.70 (13.17)	47.95 (26.23)	0.328	0.21	(0.00, 1.00)
	% RDI	262.50 (102.67)	199.20 (47.55)	225.78 (51.14)	311.33 (87.81)	353.77 (223.12)	0.326	0.21	(0.00, 1.00)
B6	Dietary intake (mg)	2.32 (0.72)	1.96 (0.26)	2.50 (0.70)	2.74 (0.96)	1.90 (0.57)	0.471	0.03	(0.00, 1.00)
	% RDI	141.39 (46.33)	115.29 (15.34)	14706 (41.18)	161.18 (56.67)	148.66 (85.46)	0.476	0.03	(0.00, 1.00)
Folic acid	Dietary Intake (µg)	299.13 (182.94)	238.20 (112.36)	218.67 (36.96)	419.60 (255.78)	271.00 (203.65)	0.578	0.00	(0.00, 1.00)
	% RDI	76.05 (44.71)	59.55 (28.09)	54.67 (9.24)	104.90 (63.95)	77.28 (37.44)	0.528	0.00	(0.00, 1.00)
B12	Dietary Intake (µg)	4.59 (2.36)	3.66 (1.79)	2.60 (0.35)	6.14 (3.66)	3.70 (1.70)	0.328	0.20	(0.00, 1.00)
	% RDI	221.45 (120.82)	183.00 (89.27)	130.00 (17.32)	307.00 (182.85)	126.39 (1.96)	0.253	0.22	(0.00, 1.00)
Vitamin C	Dietary intake (mg)	146.81 (137.31)	117.32 (82.61)	119.27 (29.95)	219.36 (214.16)	80.50 (98.29)	0.796	0.00	(0.00, 1.00)
	% RDI	210.18 (185.66)	160.32 (109.179	174.32 (41.86)	320.75 (282.57)	112.22 (124.14)	0.719	0.00	(0.00, 1.00)
Vitamin A	Dietary Intake (µg)	955.00 (633.98)	577.80 (289.47)	573.33 (112.51)	1661.40 (607.74)	704.50 (142.13)	0.088	0.52	(0.00, 1.00)
	% RDI	135.11 (92.80)	78.00 (39.88)	70.63 (22.12)	241.80 (80.52)	107.83 (10.14)	0.028	0.62	(0.00, 1.00)
Vitamin D	Dietary Intake (µg)	2.27 (2.30)	1.29 (1.34)	1.39 (1.06)	4.27 (2.94)	1.00 (0.28)	0.464	0.00	(0.00, 1.00)
	% RDI	45.35 (46.09)	25.84 (26.79)	27.87 (21.11)	85.44 (58.78)	20.10 (5.52)	0.269	0.18	(0.00, 1.00)
Vitamin E	Dietary intake (mg)	8.53 (5.00)	5.65 (2.29)	7.40 (2.44)	13.36 (5.75)	5.35 (1.48)	0.184	0.33	(0.00, 1.00)
	% RDI	56.84 (33.33)	37.60 (15.24)	49.33 (16.29)	89.07 (38.36)	35.67 (9.90)	0.184	0.33	(0.00, 1.00)
Vitamin K	Dietary Intake (µg)	153.23 (172.33)	122.48 (131.60)	60.07 (17.29)	201.36 (247.47)	249.50 (200.11)	0.473	0.02	(0.00, 1.00)
	% RDI	174.25 (190.98)	136.09 (146.22)	66.74 (19.21)	223.73 (274.97)	307.22 (179.92)	0.366	0.16	(0.00, 1.00)
Retinol	Dietary Intake (µg)	458.88 (370.58)	259.54 (182.12)	244.67 (61.46)	679.40 (591.27)	523.00 (62.23)	0.028	0.67	(0.00, 1.00)
	% RDI	70.60 (57.01)	38.89 (32.25)	37.64 (9.46)	117.14 (75.99)	80.46 (9.57)	0.366	0.16	(0.00, 1.00)
Betacarotene	Dietary Intake (µg)	1967.13 (1182.96)	1585.40 (798.94)	1751.00 (895.26)	2676.00 (1694.99)	1473.50 (273.65)	0.602	0.00	(0.00, 1.00)
	% RDI	281.002 (168.99)	226.49 (114.13)	250.14 (127.89)	382.29 (242.14)	210.50 (39.09)	0.602	0.00	(0.00, 1.00)

## Data Availability

There are restrictions on the availability of data for this trial due to the signed consent agreements around data sharing, which only allow access to external researchers for studies following the project’s purposes. Requestors wishing to access the trial data used in this study can make a request to mariscal@ugr.es.
